# External Exposure to BTEX, Internal Biomarker Response, and Health Risk Assessment of Nonoccupational Populations near a Coking Plant in Southwest China

**DOI:** 10.3390/ijerph19020847

**Published:** 2022-01-13

**Authors:** Ning Qin, Yuanyuan Zhu, Yan Zhong, Jing Tian, Jihua Li, Laiguo Chen, Ruifang Fan, Fusheng Wei

**Affiliations:** 1 School of Energy and Environmental Engineering, University of Science and Technology Beijing, Beijing 100083, China; qinning@ustb.edu.cn (N.Q.); weifsh@cae.cn (F.W.); 2 China National Environmental Monitoring Center, Beijing 100012, China; 3 Anshan Ecological Environment Monitoring Center of Liaoning Province, Anshan 114000, China; zysonice@163.com (Y.Z.); tianjing751229@126.com (J.T.); 4 Qujing Center for Disease Control and Prevention, Qujing 655011, China; ynqj_cn@sina.com; 5 Key Laboratory of Water and Air Pollution Control of Guangdong Province, South China Institute of Environmental Science, Ministry of Ecological Environment, Guangzhou 510655, China; chenlaiguo@scies.org; 6 Air Pollution Control Engineering Laboratory of Guangdong Province, South China Institute of Environmental Science, Ministry of Ecological Environment, Guangzhou 510655, China; 7 Guangzhou Key Laboratory of Subtropical Biodiversity and Biomonitoring, School of Life Science, South China Normal University, Guangzhou 510631, China; tgxb@jnu.edu.cn

**Keywords:** benzene homologues, biomarkers, external exposure, health risk

## Abstract

Benzene, toluene, ethylbenzene and xylene isomers (BTEX) have raised increasing concern due to their adverse effects on human health. In this study, a coking factory and four communities nearby were selected as the research area. Atmospheric BTEX samples were collected and determined by a preconcentrator GC–MS method. Four biomarkers in the morning urine samples of 174 participants from the communities were measured by LC–MS. The health risks of BTEX exposure via inhalation were estimated. This study aimed to investigate the influence of external BTEX exposure on the internal biomarker levels and quantitatively evaluate the health risk of populations near the coking industry. The results showed that the average total BTEX concentration in residential area was 7.17 ± 7.24 μg m^−3^. Trans,trans-muconic acid (T,T-MA) was the urinary biomarker with the greatest average level (127 ± 285 μg g^−1^ crt). Similar spatial trends can be observed between atmospheric benzene concentration and internal biomarker levels. The mean values of the LCR for male and female residents were 2.15 × 10^−5^ and 2.05 × 10^−5^, respectively. The results of the risk assessment indicated that special attention was required for the non-occupational residents around the area.

## 1. Introduction

BTEX (benzene, toluene, ethylbenzene, m-, p- and o-xylenes) are a series of volatile organic compounds (VOCs) with high detection frequency in environmental media and adverse effects on human health [1,2,3]. Exposure to BTEX can cause a variety of health problems [4,5] including adverse effects on the nervous system, immune functions and reproductive outcome in humans [6,7,8,9]. Even worse, epidemiological studies and case reports provide clear evidence of a causal association between benzene exposure and leukemia [10,11,12]. Therefore, BTEX have raised increasing public concern in recent years and benzene has been listed as a group 1 carcinogen by the International Agency for Research on Cancer [13].

BTEX are released from a variety of sources, including combustion of wood and fuels, traffic, industrial paints, adhesives, degreasing agents and aerosols. Coke production is an important contributor of environmental BTEX, accounting for more than 10% of the total industrial VOCs emissions in China [14,15,16]. Therefore, BTEX released by the coking industry may pose a threat to not only the occupational population in the factory but also the nonoccupational population living around.

External exposure estimation and internal biomarker monitoring are two major assessment methods to evaluate the health risk of BTEX. External exposure models have been established by USEPA and conducted in an enormous amount of research [16,17,18]. However, the accuracy of external exposure models has long been questioned because they are highly simplified. The bioavailability of chemicals is not considered in the assessment, which brings great uncertainty in the risk estimation. Therefore, in recent years, more attention has been paid to the biomarkers of BTEX in body fluids [19,20,21,22,23]. A biomarker of exposure is defined as a xenobiotic substance or its metabolites or the product of an interaction between a xenobiotic agent and some target molecules or cells [24]. For instance, the initial metabolic step involves the CYP oxidation of benzene to benzene oxide, which exists in equilibrium with its tautomer oxepin. Benzene oxide can spontaneously rearrange to phenol, be further metabolized to hydroquinone and 1,4-benzoquinone or be hydrolyzed to produce catechol and 1,2-benzoquinone, or reacts with glutathione to produce S-phenylmercapturic acid (S-PMA). The metabolism of oxepin is thought to open the aromatic ring, yielding the reactive muconaldehydes and trans,trans-muconic acid (T,T-MA) [25]. Because the levels of biomarkers are directly related to the human internal exposure levels, biomarkers detected from human body fluids are believed to better reflect the impact of chemicals on the human body [26]. T,T-MA and S-PMA are reported as benzene exposure indicators [27,28,29]. Significant exposure–response relationship has been reported between the external BTEX exposure and urinary biomarker concentrations. However, most of the previous research studies were focused on the occupational population [23]. The research on exposure–response relationship in low-dose exposure populations is scarce. Further research should be conducted on nonoccupational populations to get a comprehensive understanding of the relationship between external and internal exposure.

**Figure 1:**
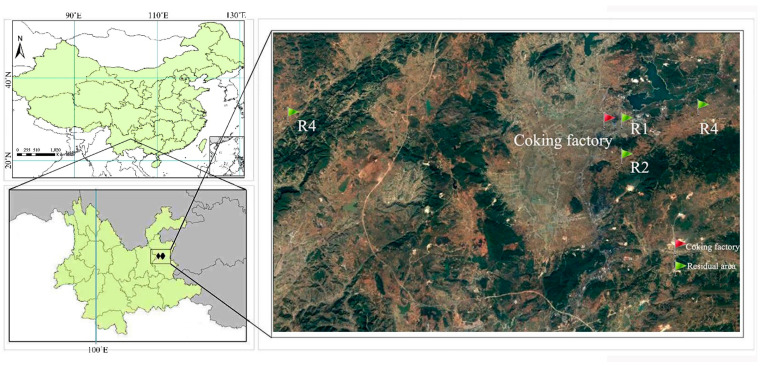
Research area and sampling sites. Atmospheric samples in communities were collected at sites R1, R2, R3 and R4.

In this study, a city located in the east of the Yunnan province (Figure 1) was selected as the research area. In recent years, the city has seen a rapid expansion in the number of coking industries due to the rapid social economic growth. Moreover, the research area has a relatively high cancer mortality rate. The Chinese age-standardized mortality rate (CASMR) of leukemia of the city was two times higher than China’s average level. Environmental exposure levels in four residential areas near a coking factory were investigated; the urinary concentration of four biomarkers from the population in the residential area were measured. This study aimed to (1) assess the external exposure level of benzene homologues through environmental contact; (2) describe the trend of environmental pollutants exposure (BTEX) with internal biomarkers; (3) try to characterize the sources of the exposure through the BTEX ratios and (4) estimate the potential health risk of BTEX exposure in nonoccupational populations.

## 2. Methods

### 2.1. Research Area and Participants

The research area was located in the east of the Yunnan province. It belongs to one of the most important economic cities in the Yunnan province. The main climate type of the study area is typical subtropical plateau monsoon. The annual mean temperature is 14.5 °C. Southwest wind is prevailing in this region. The average annual precipitation is around 1000 mm [30]. 

A coking factory with annual production capacity over three million tons was selected in this research. The land around the coking plant is mainly farmland. Three communities (R1, R2, R3) are located downwind of the factory. A residential area upwind (R4) was selected as the background site. The distances of regions R1, R2, R3 and R4 to the coke factory were 1.3 km, 3.0 km, 7.5 km and 25.0 km, respectively. The research area and sampling sites are illustrated in Figure 1.

A total of 174 nonoccupational participants were recruited within the four communities. As the major potential polluted area, 50 participants’ samples were collected in all regions R1, R2 and R3. At site R4, 25 samples were collected as a control group. Participants with genetic diseases, lung cancer and other diseases as well as occupational workers in the coking factory were excluded. Each participant was interviewed by trained recruiters. A consent form was signed by each participant before the questionnaire survey and sampling. Participants for the questionnaire survey and morning urine sampling were randomly selected from each study site, which covered all age groups (20–69 years) and genders.

A questionnaire survey was conducted among participants according to the population age structure and sex ratio of the four regions. All participants had lived in their local regions for 18.9 years on average, and information about their individual characteristics, lifestyle factors, job information, smoking, cooking and dietary habits was collected. The smoking rate of adult females was close to zero. The questionnaire survey showed that there was no significant difference in people’s lifestyle and cooking habit in the four regions. The basic information of participants is shown in Table 1.

### 2.2. Sampling and Instrumental Analysis

Atmospheric BTEX samples were collected from four residential regions using stainless steel canisters (3.2 L, Silonite™ Canisters, Entech Instruments Inc., Simi Valley, CA, USA) from 15 October to 20 October. In order to quantitatively compare the difference between occupational and nonoccupational exposure levels, samples were also collected at seven sites inside the factory including two sampling sites in the offices, two on the top of coke ovens, one sample next to the chimney, one in the coal charging workshop and one in the coke discharging workshop. A total of five samples were collected at different times at each sampling site during the sampling period. The morning urine samples of 174 adults were collected on 23 October 2012. 

Atmospheric BTEX samples were determined by a preconcentrator GC–MS method, which has been used in previous research [31,32]. A total of 300 mL samples and 50 mL internal standard gas (1,4-difluorobenzene) were concentrated in the Model 7100 preconcentrator (Entech Instruments Inc., Simi Valley, CA, USA), and measured by a gas chromatography-mass selective detector (GC-MSD/FID, Agilent 7890A/5975C, USA).

The urine samples were extracted using a solid phase extraction (SPE) system (C18 SPE cartridge, 500 mg, 6 mL, Varian, CA, USA). Urine samples were analyzed by an Agilent 6460 LC–MS triple quadrupole system (Santa Clara, CA, USA) with an Ultra HPLC 1290, G4220A Infinity Binary Pump, G1316C Infinity TCC, and a G4226A Infinity Sampler. Details of the analytical and quantification procedures have been previously described [21]. Four internal biomarkers were measured including T,T-MA, S-PMA, 1,2-dihydroxybenzene (1,2-DB) and S-benylmercapturic acid (S-BMA). The urinary creatinine concentrations were determined with a Hitachi 7600-110 automatic biochemistry analyzer (Hitachi, Japan). Creatinine is a natural metabolic product of creatine that occurs in muscle tissue and an approximately constant amount of creatinine is produced daily. It is believed that biomarker production or excretion has a linear relationship with biomarker excretion across individuals. Therefore, creatinine can be used to adjust the biomarker level to eliminate the influence of urinary flow rate [33].

QA/QC was conducted using field and laboratory blanks, duplicate samples, standard spiked recoveries, relative standard deviations (RSDs) and method detection limits (MDLs). The method recoveries in air and urine samples were within the acceptable range of 70% to 130%. The recovery of six benzene homologues were from 75.2% to 102.3% (Table S1). The RSDs of samples were below 20%. 

### 2.3. Risk Assessment

The noncarcinogenic risk and carcinogenic risk through BTEX inhalation were estimated. In addition, two biomarkers, T,T-MA and S-PMA, levels in urine samples of local residents were compared with biological exposure indices (BEIs) derived by the American Conference of Governmental Industrial Hygienists (ACGIH) to assess the internal benzene exposure risk. An assessment of the toxicity of noncarcinogens is based on the concept of threshold below which no adverse health effects can be observed [34]. The hazard quotients (HQ) of noncarcinogenic BTEX were calculated using the following equation,

(1) $$\mathrm{HQ}=\frac{C}{\mathrm{RfC}}$$

where RfC refers to the threshold values. For BTEX inhalation, RfC is the inhalation reference concentration (mg m^−3^). For benzene metabolites, the RfC values are the BEIs derived by ACGIH. Detrimental health effects are unlikely when HQ < 1, whereas there may be cause for concern from potential noncarcinogenic health risks when HQ > 1. 

The estimates of carcinogenic risks are reported as lifetime cancer incidence risks (LCRs). The LCR of the studied population related to benzene inhalation was estimated using the following equation:

(2) LCR=CDI×SF

where CDI is the chronic daily intake of benzene (mg kg^−1^ day^−1^) and SF is the cancer slope factor. SF is basically the slope of the dose–response curve at very low exposures. In this study, SF was 0.029 mg kg^−1^ day^−1^ [35]. The CDI was calculated as:

(3) $$\mathrm{CDI}=\frac{C\times \mathrm{IR}\times \mathrm{EF}\times \mathrm{ED}}{\mathrm{BW}\times \mathrm{AT}}$$

where C is the contaminant concentration (μg m^−3^); IR is the inhalation rate (m^3^ day^−1^); EF is the exposure frequency (365 day year^−1^); ED is the exposure duration; in this study, we focus on the adults (18–70 years old) in the research area and the ED was therefore 53 years; BW is the body weight (kg); and AT is the averaging time (25,550 days). The chemical concentrations, inhalation rate and body weight are considered as variables and obtained from monitoring and literature research.

### 2.4. Derivation of Exposure Parameters

**Figure 2:**
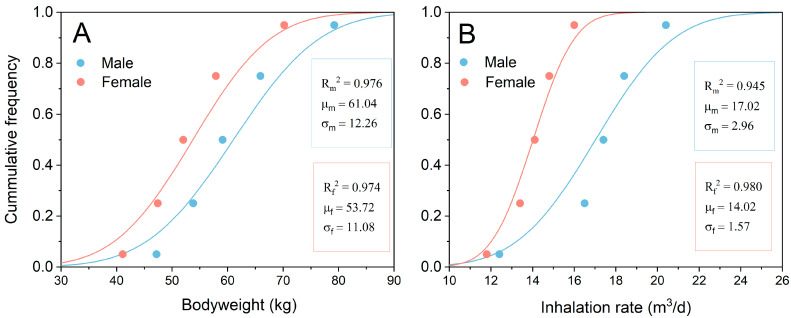
Fitting of bodyweight (**A**) and inhalation rate (**B**) of male and female population in Yunnan province. μ and σ are the arithmetic mean and standard variation of the normal distribution, respectively.

Parameters used in the risk assessment were obtained by both experimental monitoring and literature research. Kolmogorov-Smirnov tests were applied to check the distribution mode. Normal and log-normal models were employed to describe the data distribution, because they can best describe the exposures in the risk assessment models [36,37,38]. The quartiles and medians of population bodyweight and inhalation rate from the Yunnan province were obtained from the Exposure Factors Handbook of Chinese Population. The weight and inhalation rate distributions of the population were fitted using the Gaussian function. The fit curves are shown in Figure 2. The parameters used in the risk assessment are shown in Table 2.

### 2.5. Statistical Analysis

The data were then processed using SPSS 21.0. A Kruskal–Wallis test and Mann–Whitney U test were used to detect the differences in biomarkers between different regions and population groups. The relationship between the data was determined using Spearman’s sample correlation, and when the value of *p* was less than 0.05, the correlation was regarded as significant. A probabilistic risk assessment method was applied. Monte Carlo simulations of 10,000 iterations were used to evaluate the risk distribution and parameter sensitivity. 

## 3. Results and Discussion

### 3.1. Atmospheric BTEX Levels 

**Figure 3:**
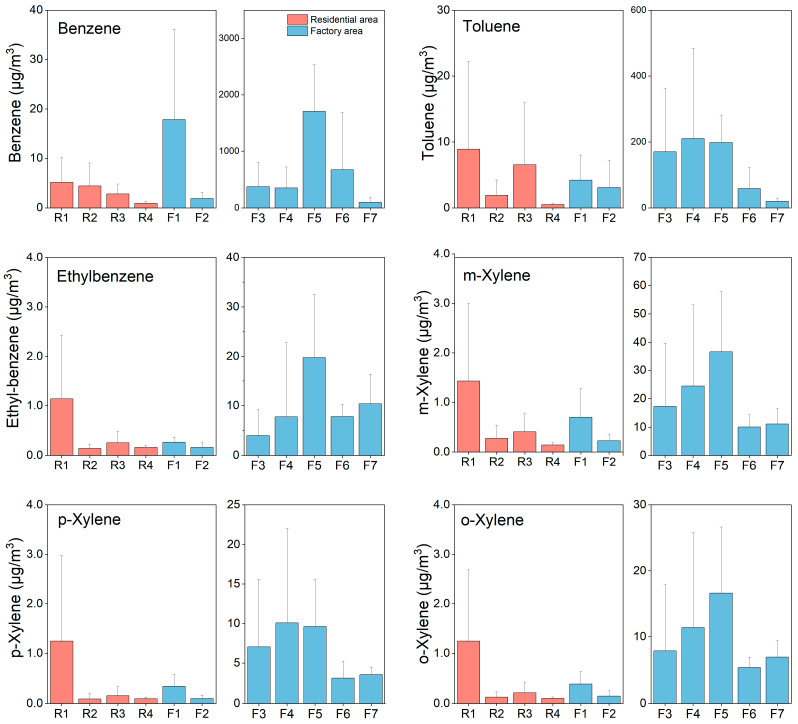
Atmospheric concentrations of benzene, toluene, ethylbenzene and xylenes in residential areas and at a coking factory. Red histograms represent the residential areas and blue ones represent sampling sites at the factory. Samples F1 and F2 were collected from two offices, F3 and F4 were from the top of two coke ovens in the factory. F5 to F7 were from chimneys, coal charging workshop and coke discharging workshop, respectively.

The levels of spatial variation of BTEX in residential areas and at the factory are illustrated in Figure 3. For residential areas, the greatest total BTEX concentration (ΣBTEX) was found at sampling site R1 with a concentration of 19.1 μg m^−3^, followed by R3 and R2 with a ΣBTEX of 10.4 and 6.9 μg m^−3^, respectively. Site R4 had the least content at 1.9 μg m^−3^. The average ΣBTEX in residential areas was 7.17 ± 7.24 μg m^−3^. Benzene and toluene were the pollutants with a higher concentration, contributing to 41.9% and 41.4% of the ΣBTEX, respectively. The concentrations of ethyl and xylene were relatively low with total contribution of 16.8% to the ΣBTEX. Compared with the BTEX concentrations reported in the other nonoccupational scenarios, ΣBTEX in our research was comparable to the value of 8.61μg m^−3^ reported in Ahvaz, Iran [45], but lower than the data reported in cities in China such as Beijing (18.5 μg m^−3^) and Guangzhou (610 μg m^−3^). It was also lower than Sao Paulo (60.5 μg m^−3^) in Brazil and Delhi (373 μg m^−3^) in India [46]. 

For the factory area, the highest total BTEX concentration was detected at site F5 (1990 μg m^−3^), followed by F6 (757 μg m^−3^) and F4 (616 μg m^−3^). The residual levels in two offices were relatively low with concentrations of 23.7 μg m^−3^ and 5.60 μg m^−3^, respectively. It can be found that workers at the coal charging workshop and near coke ovens may suffer from a higher BTEX exposure than those in offices. Compared with BTEX concentrations in the other occupational exposure scenarios reported abroad, the concentration in this research was much higher than data reported at a gas station in Porto, Portugal with BTEX concentration 15.3 μg m^−3^, comparable with tank-gauging workers (830 μg m^−3^), but higher than drivers (81.9 μg m^−3^), firefighters (71.2 μg m^−3^) and office workers (19.8 μg m^−3^) in Iran [47].

### 3.2. Profiles of Urinary BTEX Metabolites

The metabolism of benzene homologues is complex. T,T-MA, S-PMA and 1,2-DB are metabolites of benzene [29], in which T,T-MA and S-PMA are widely reported as biomarkers of high/low levels of exposure to benzene, respectively [48,49]. S-BMA is reported as a toluene metabolite in human urine [50]. For the metabolism of ethylbenzene via inhalation, around 90% of the adsorbed dose is excreted as mandelic acid and phenylglyoxylic acid [51]. The amount of methyl hippuric acid in urine has been recommended as an indicator of occupational exposure to xylene [52]. In this research, T,T-MA, S-PMA, 1,2-DB and S-BMA were selected as target biomarkers because of the high residual levels of atmospheric benzene and toluene in our research. The biomarker concentrations were corrected by creatinine (crt) concentration to make them comparable among individuals [53]. The crt-corrected urinary biomarker concentrations of the population from the four regions are shown in Table 3. T,T-MA had the highest concentration with an average value of 127 ± 285 μg·g^−1^ crt, followed by 1,2-DB with a content of 64.8 ± 95.2 μg·g^−1^ crt. S-PMA had the lowest concentration.

The four BTEX metabolites were measured in occupational exposure population in the factory area at the same time by previous research [54]. The T,T-MA level in our study was lower than the level of occupational workers (161 ± 174 μg·g^−1^ crt). However, S-PMA (2.29 ± 2.32 μg·g^−1^ crt) and S-BMA (5.01 ± 6.99 μg·g^−1^ crt) in our study and previous report were comparable. The 1,2-DB level was higher than that of workers in the factory (35 ± 125 μg·g^−1^ crt). It seems that the differences of biomarkers were not significant between occupational and nonoccupational workers. Compared with nonoccupational population in China and abroad, the concentration of T,T-MA was lower than urban residents reported in Iran (275 ± 57 μg·g^−1^ crt) and comparable to the levels in a rural area (160 ± 52 μg·g^−1^ crt) [55]. The S-PMA level in our research was also higher than in children (0.68 ± 1.72 μg·g^−1^ crt) and nonsmoking elderly people (0.49 ± 1.30 μg·g^−1^ crt) in the Shanxi province, but lower than smoking elderly people (3.04 ± 4.62 μg·g^−1^ crt) in high-exposure scenario [56].

Kruskal–Wallis and Mann-Whitney U tests were applied to compare the differences caused by spatial variation, gender and smoking. For all four BTEX metabolites, significant spatial differences were detected (Table 3). The results also indicated that the gender difference was not significant (*p* > 0.05). In addition, smoking is supposed to be a pathway human exposure to BTEX. It has been estimated that smokers receive about 90% of their benzene intake from smoking [57]. It has also been reported that T,T-MA and S-PMA measured in smokers were even higher than in occupational populations [58]. From the result of the Kruskal–Wallis test, however, a significant difference was only detected in levels of 1,2-DB, with a *p* value of 0.021. No significant variation was found in levels of T,T-MA (*p* = 0.935), S-PMA (*p* = 0.058) and S-BMA (*p* = 0.772) between smokers and nonsmokers. Low-exposure concentration can be a potential reason accounting for the results.

The spatial trends of atmospheric BTEX levels and biomarkers were compared to analyze the relationship between external exposure and internal biomarkers. The geomean of atmospheric benzene concentration in four communities, from high to low was: R1 (5.14 μg m^−3^), R2 (4.41 μg m^−3^), R3 (2.83 μg m^−3^) and R4 (0.91 μg m^−3^), which is consistent with the order of S-PMA content. The geomean of T,T-MA from high to low was: R1, R2, R4 and R3; and the geomean of 1,2-DB from high to low was: R1, R3, R2 and R4. Similar trends can be found in the spatial distribution of metabolites of benzene despite some small differences. Participants in R1, which was also the nearest site to the coking plant, had the highest levels of the four biomarkers, which indicated a potential influence of atmospheric benzene on the internal biomarkers. 

### 3.3. Sources Apportionment of BTEX

**Figure 4:**
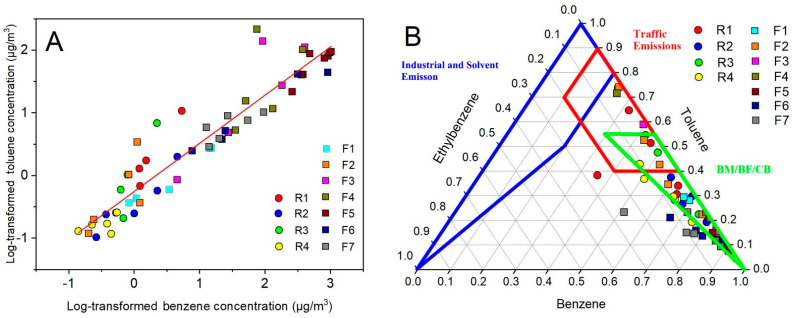
(**A**) Correlation between benzene and toluene and (**B**) source apportionment of BTEX from eleven sampling sites. Areas in green, blue and red outlines are profiles of biomass/biofuel/coal burning (BM/BF/CB), industrial and solvent emissions and traffic emissions, respectively.

A good correlation between homologues indicates that the primary source of origin for these pollutants is similar [45]. Otherwise, the local BTEX is likely to come from different pollution sources. The relationship between benzene and toluene [15] is the most widely used indicator due to their relative high detection rates. A Spearman correlation was employed to study the benzene and toluene concentrations at the sampling sites. The result is illustrated in Figure 4A. The closer the points are to the line, the more likely they are to be from the same source. It can be seen that although the concentration at eleven sites varied greatly, most of the sampling sites were scattered close to the line. The result indicated that the local BTEX sources were not complex.

The ratios between BTEX species are important indicators to provide information about the different emission sources of these pollutants [59,60,61]. For example, the toluene/benzene ratios were <1 for biomass/biofuel/coal burning, 1–10 for vehicle emissions, and >1 for industrial processes and solvent applications [31]. The amount of (m-xylene + p-xylene)/ethylbenzene ratio was approximately 3.6 in urban areas regardless of geographic location [62]. However, some work has reported that overlapping ratios exist among different sources. Sometimes, the ratio of the same source may also vary greatly. Therefore, it is very important to clarify the ratio characteristics of different sources before using them to explore the sources [31]. Zhang studied the concentration profiles of more than 230 samples from eight typical BTEX sources in China and established a method that analyzed the potential sources through the relative compositions of benzene, toluene, and ethylbenzene [63]. The proportions of B, T, and E from atmospheric sampling sites are marked in Figure 4B. The range of different sources is also circled in different colors. It can be found that most of the samples were scattered within the composition ranges for biomass/biofuel/coal burning. The results were consistent with the environment of the coking plant. We can also see the influence of traffic emissions on many sampling sites, indicating that vehicle-related emissions also had a certain contribution to the BTEX in the area. In contrast, the distribution of sample points in the figure shows that industrial process solvent volatilization had a limited impact on both sampling sites in and out of the factory. One possible reason is that coking is a special kind of industry, which uses coal as raw material. Therefore, the emission profile may be closer to coal combustion instead of industrial emission.

### 3.4. Health Risk of Population in Residential Area

#### 3.4.1. Noncarcinogenic Risk

The RfCs of BTEX, the BEIs of two benzene metabolites and noncancer HQs of different sampling sites are shown in Table 4 [41,64]. The HQs of four chemicals cannot be compared because the toxicity endpoint of chemicals was different. For example, toxic effects are first reflected in the immune system for benzene, whereas the target of toluene and xylene is the nervous system. The ACGIH has established 25 μg S-PMA/g crt and 500 μg T,T-MA/g crt in the urine as BEIs for benzene exposure in the workplace. It should be noted that the BEI is primarily an index of exposure and not a level at which health effects might occur from exposure to benzene. BEI values were applied in our research to assess the internal exposure levels for nonoccupational population.

**Figure 5:**
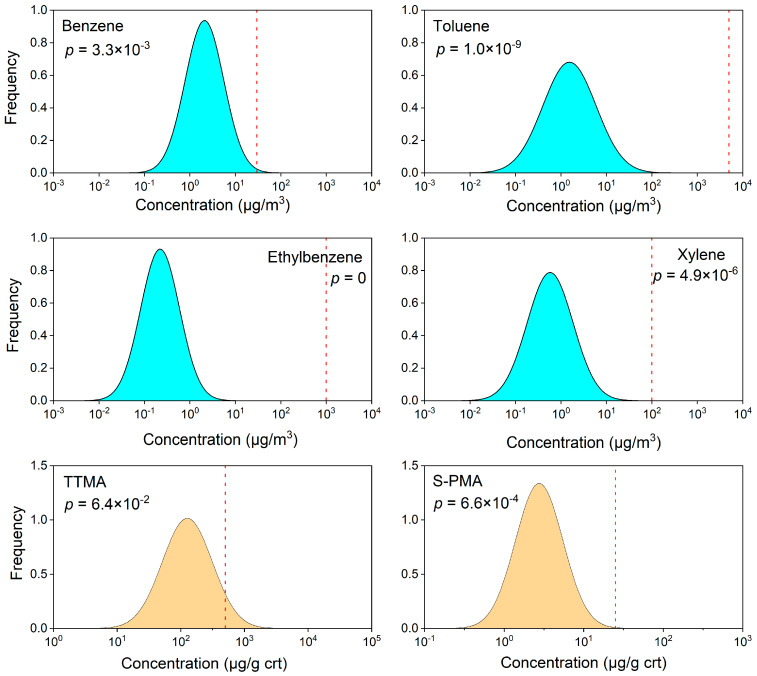
Distribution of atmospheric BTEX concentrations in residential areas and urinary biomarker concentrations from residents. Vertical lines represent noncancer risk threshold reference concentrations.

It was found that the HQs in residential areas were lower than 1, which indicated a relatively low noncarcinogenic risk. In general, the HQs of benzene and T,T-MA were similar. The risk values derived by S-PMA were quite close, especially in R2, R3 and R4. In the factory area in contrast, the HQs of benzene in F3 to F7 were much higher than 1. It means a high noncancer risk especially in F6 (coal charging workshop). In order to provide a clear picture of noncancer risk, a probabilistic risk assessment was applied. The distributions of chemical concentrations were compared with the RfCs and BEIs and the probability exceeding the threshold levels (*p*) was estimated (Figure 5).

It can be seen that the population in the area has the highest probability to exceed the threshold of benzene. A probability of 0.3% of adults may suffer benzene exposure higher than the RfC. According to the HQ of T,T-MA, the probability of urinary T,T-MA exceeding the BEI derived to protect occupational workers was 6.4%.

There is a slight difference between the risk estimated based on T,T-MA and S-PMA. T,T-MA and S-PMA are both considered as biomarkers of exposure to benzene. However, it was also reported that urinary levels of T,T-MA do not always correlate well with the air concentration of benzene because it is also a metabolite of sorbic acid, a common food additive [55]. In contrast, S-PMA has been considered to be a sensitive and specific biomarker for evaluating benzene exposure at low levels. Therefore, using T,T-MA as a marker may lead to overestimation of the internal exposure level when the benzene exposure level is low. For all the benzene homologues, the probability of noncarcinogenic risk of benzene was much higher than that of TEX.

#### 3.4.2. Carcinogenic Risk

Epidemiological evidence suggests that exposure to benzene has been associated with the development of a particular type of leukemia called acute myeloid leukemia. Our research area has a high leukemia mortality rate [65]. A retrospective death survey covering a 7-year population of 370,195 person-years from 2005 to 2011 was conducted to determine the mortality rate of cancer. The Chinese age-standardized mortality rate (CASMR) was studied in the exposure area (a combination of residential area R1, R2 and R3) and background area (R4). The leukemia mortality rate in the exposure area and background area were 9.27 per 100,000 person-years and 4.56 per 100,000 person-years, which were higher than China’s average level (3.85 per 100,000 person-years) [66].

**Figure 6:**
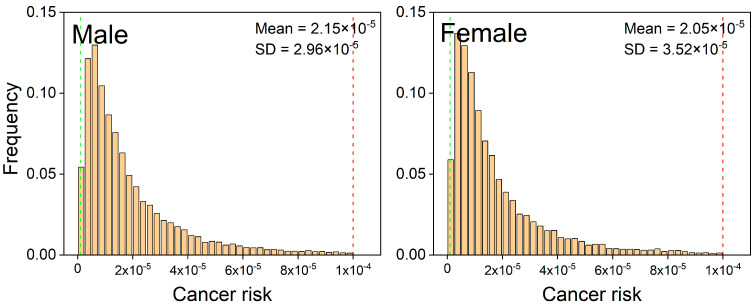
Distributions of lifetime cancer risk for male and female population groups derived using Monte Carlo simulations. Green and red vertical lines represent the US EPA acceptable risk level and serious risk level, respectively.

The distributions of lifetime cancer risk for male and female population groups were derived using Monte Carlo simulation (Figure 6). The mean value of the LCR for male and female residents was 2.15 × 10^−5^ and 2.05 × 10^−5^, respectively, which shows a noticeably higher carcinogenic risk for adult males than for female residents. This result is consistent with the survey of death causes. The study also found male residents had a significantly higher mortality from leukemia (12.44 per 100,000 person-years) than female residents (5.36 per 100,000 person-years) in the area [66].

According to the criteria suggested by the US EPA, a one-in-a-million chance of an additional human cancer over a 70-year lifetime is an acceptable level of risk and a one-in-ten-thousand or greater chance is considered to be a serious risk. It can be found that almost the entire population within the research area exceeded acceptable limits. Moreover, approximately 2.1% of male residents and 1.8% of female residents may suffer a carcinogenic risk higher than the serious risk level. The results of health risk assessment indicated a potential carcinogenic risk of nonoccupational residents living in the communities nearby. Special attention is therefore required for not only the occupational population in the factory, but also for the residents around the area.

### 3.5. Limitations

One limitation of the current study is the small sample size. A total of 50 participants were selected to represent the population in each residential area due to the time and cost. Efforts have been taken to reduce the influence of the sample size. The population characteristics were well investigated, and the gender and age structure of the participants were analyzed. The subjects were considered to be representative and the impact of the sample size is likely to be small. Another limitation is the sampling time of morning urine. The metabolic time of benzene series varies from person to person; therefore, it is difficult to collect urine samples accurately corresponding to atmospheric samples. Because the discharge of the factory was stable during our sampling, we believe that the external BTEX exposure level and the biomarker levels in morning urine remained stable. Finally, not all the metabolites of BTEX were investigated in this research. More attention is required for the metabolism and health risk of ethylbenzene and xylenes in future research.

## 4. Conclusions

This study estimated the health risk of nonoccupational residents near a coking factory based on external inhalation exposure levels and internal biomarker levels. The results of source apportionment indicated biomass or coal burning was the major source in the research region. A potential association can be observed between atmospheric benzene concentration and internal biomarker levels. According to the probabilistic risk assessment, the probability of atmospheric benzene content exceeding the RfC was 0.3% and the probability of urinary T,T-MA concentration exceeding the BEI was 6.4%. For cancer risk, approximately 2.1% of male and 1.8% of female residents may suffer carcinogenic risk higher than the serious risk level recommended by the US EPA. Further strategies need to be formulated to control the BTEX emission and reduce the potential health risk for nonoccupational populations nearby.

## Figures and Tables

**Table 1:** Basic information on research area.

Category	R1	R2	R3	R4
Distance to the coking plant	1.3 km	3.0 km	7.5 km	25.0 km
Function division	Coke industry, residential area, hospital, school and commercial area	Residential area, farmland, reservoir and wetland	Residential area and farmland	Residential area and farmland
Number of residents	5598	1525	3115	2453
Sample size	50	49	50	25
Male	25	25	24	11
Female	25	24	26	14
Age structure				
20–30	13	11	15	4
31–40	17	13	10	7
41–50	8	14	13	4
51–60	9	9	7	8
61–70	3	2	4	2
Smokers (male)	68.0%	72.0%	62.5%	81.8%

**Table 2:** Parameters of risk assessment.

Parameters	Unit	Threshold Values	Distribution	Parameter *a*	Parameter *b*	Reference
Benzene	μg·m^−3^	30 [39]	Log-normal	0.321	0.426	Measured
Toluene	μg·m^−3^	5000 [40]	Log-normal	0.188	0.586	
Ethyl-benzene	μg·m^−3^	1000 [41]	Log-normal	−0.655	0.428	
(m+p+o)-xylene	μg·m^−3^	100 [42]	Log-normal	−0.238	0.506	
T,T-MA	μg·(g·crt)^−1^	500 [43]	Log-normal	2.102	0.392	
S-PMA	μg·(g·crt)^−1^	25 [43]	Log-normal	0.441	0.298	
BW-male	kg		Normal	61.04	12.26	Exposure Factors Handbook of Chinese Population [44]
BW-female	kg		Normal	53.72	11.08	
IR-male	m^3^·day^−1^		Normal	17.02	2.96	
IR-female	m^3^·day^−1^		Normal	14.02	1.57	

For a normal distribution, parameter *a* is the mean value, parameter *b* is the standard deviation. For a log-normal distribution, parameter *a* is the mean of log-transformed values, parameter *b* is the standard deviation of log-transformed values.

**Table 3:** BTEX metabolite concentrations (μg·g^−1^ crt) and spatial differences by Kruskal–Wallis test (GM ± SD).

	R1 (N = 50)	R2 (N = 49)	R3 (N = 50)	R4 (N = 25)	Kruskal–Wallis Test
T,T-MA	213 ± 434	128 ± 197	83 ± 164	98.1 ± 75.8	*p* < 0.01
1,2-DB	91.4 ± 73.4	58 ± 121	62.8 ± 65.9	43 ± 120	*p*< 0.01
S-PMA	4.23 ± 7.68	2.35 ± 1.29	2.30 ± 1.49	2.06 ± 0.76	*p* < 0.01
S-BMA	7.98 ± 7.88	4.7 ± 10.9	6.90 ± 4.80	7.6 ± 13.9	*p* < 0.05

**Table 4:** Noncancer risk (HQ) of sampling sites in residential and factory areas.

Chemicals		Benzene	Toluene	Ethylbenzene	Xylene	T,T-MA	S-PMA
Residential area	R1	0.171	0.002	0.001	0.039	0.43	0.17
	R2	0.147	0.000	0.000	0.005	0.26	0.09
	R3	0.094	0.001	0.000	0.008	0.17	0.09
	R4	0.030	0.000	0.000	0.003	0.20	0.09
Factory area	F1	0.595	0.001	0.000	0.014		
	F2	0.063	0.001	0.000	0.005		
	F3	12.431	0.034	0.004	0.321		
	F4	11.730	0.042	0.008	0.460		
	F5	56.963	0.040	0.020	0.628		
	F6	22.411	0.012	0.008	0.185		
	F7	3.263	0.004	0.010	0.216		

## Data Availability

Not applicable.

## Supplementary Materials

The following supporting information can be downloaded at: https://www.mdpi.com/article/10.3390/ijerph19020847/s1, Text S1: Sampling; Text S2: Pretreatment and instrumental analysis; Table S1: Recoveries, method detection limits (MDLs) and relative standard deviation of BTEX in atmospheric samples.
