# Supplementary material for: External Exposure to BTEX, Internal Biomarker Response, and Health Risk Assessment of Nonoccupational Populations near a Coking Plant in Southwest China
Source: Int J Environ Res Public Health. 2022 Jan 13;19(2):847. doi: 10.3390/ijerph19020847 (PMC8775548; doi:10.3390/ijerph19020847)
Supplement: Supplementary file 1 [file ijerph-19-00847-s001.zip › ijerph-1514550-supplementary.pdf]

External Exposure to BTEX, Internal Biomarker Response, and Health Risk Assessment of Nonoccupational Populations near a Coking Plant in Southwest China

Ning Qin [1,*], Yuanyuan Zhu [1,2,*], Yan Zhong [3], Jing Tian [3], Jihua Li [4], Laiguo Chen [5], Ruifang Fan [6], Yulong Yan [7], Fusheng Wei [1,2]

[1] School of Energy and Environmental Engineering, University of Science and Technology Beijing, Beijing 100083, China

[2] China National Environmental Monitoring Center, Beijing 100012, China

[3] Anshan Environmental Monitoring Center, Anshan 114000, China

[4] Qujing Center for Disease Control and Prevention, Qujing 655011, China

[5] Urban Environment and Ecology Research Center, South China Institute of Environmental Sciences (SCIES), Ministry of Ecology and Environment, Guangzhou 510655, China

[6] Key Laboratory of Ecology and Environmental Science in Guangdong Higher Education, School of Life Science, South China Normal University, Guangzhou 510631,China

[7] School of Environment and Safety, Taiyuan University of Science and Technology, Taiyuan 030024, China

* Correspondence: zhuyy@cnemc.cn

Supplementary caption

Supplementary caption

Number SI pages: 5

Number the tables: 1

This supporting information provides text and tables addressing

List of texts

Text S1: Sampling

Text S2: Pretreatment and instrumental analysis

List of tables

Table S1. Recoveries, method detection limits (MDLs) and relative standard deviations (RSDs) of BTEX in atmospheric samples

Text

Text S1: Sampling

Stainless steel canisters (3.2L, Silonite™ Canisters, Entech Instruments Inc., CA) were used to collect the samples. Before sampling, the steel canisters were cleaned with high-purity nitrogen at 50 °C and evacuated to < 50 mTorr. Air was pulled down into the canister through a 1-h valve (CS1200, Entech, USA), which was calibrated with a soap bubble meter at 90 mL/min before providing a steady airflow. Samples were collected twice a day for three days at a height of 1.4 m. The sampling time was from 9:00 to 10:00 in the morning and from 15:00 to 16:00 in the afternoon for two days. The filled canisters were shipped to the laboratory for analysis within 24 h.

The first morning urine samples were collected on the morning of October 23, 2012. A total of 174 adult subjects were recruited. Subjects with genetic diseases, lung cancer, and other diseases were excluded. Each participant was interviewed by two trained recruiters. The urine samples were collected in screw-capsuled plastic bottles and shipped frozen in dry ice to the laboratory within 2 h and then stored at −20 °C before analysis.

Text S2: Pretreatment and instrumental analysis

Atmospheric BTEX samples were determined by the Preconcentrator-GC-MS method. A total of 300 mL samples and 50 mL of internal standard gas (1,4-difluorobenzene) were concentrated in the Model 7100 preconcentrator (Entech Instruments Inc., USA), and transferred into a gas chromatography-mass selective detector (GC-MSD/FID, Agilent 7890A/5975C, USA). A HP-1 capillary column (60 m × 0.32 mm × 1.0 mm, Agilent Technologies, USA) was used to separate the mixture, which was then split into two ways: one is to a PLOT-Q column (30 m × 0.32 mm × 2.0 mm, Agilent Technologies, USA) followed by FID for detecting C_2-C_3 hydrocarbons; another is

to a 80 cm × 0.10 mm I.D. stainless steel line followed by MSD for C_4-C_{12} hydrocarbons. The MSD was used in selected ion monitoring mode, and the ionization method was electron impacting (EI). Target compounds were identified based on their retention times and mass spectra.

A total of 3 mL of HAC buffer (0.1%) was added to 2 mL urine samples to adjust the pH to 4.5. Urine samples were extracted using a solid phase extraction (SPE) system (C18 SPE cartridge, 500 mg, 6 mL, Varian, CA, USA). The cartridges were eluted with 4 mL of acetonitrile. The volume of the extracts was concentrated by nitrogen and was adjusted to a volume of 100 μL. Internal standards were added for GC analysis. Urine samples were analyzed using an Agilent 6460 LC-MS Triple Quadrupole system (Santa Clara, CA, USA) with an Ultra HPLC 1290, G4220A Infinity Binary Pump, G1316C Infinity TCC, and a G4226A Infinity Sampler. The mobile phases were 0.1% HAC in water and methanol (HPLC grade Sigma-Aldrich, USA). Four metabolites, trans,trans-muconic acid (t, t-MA), 1, 2-dihydroxybenzene (1, 2-DB), S-phenylmercapturic acid (S-PMA), and S-benzylmercapturic acid (S-BMA) were separated using a Zorbax Eclipse plus phenyl–hexyl (narrow bore RRHT, 600 bar, 4.6 × 100 mm, 1.8 mm, Agilent USA). The internal standard included D_4-t,t-MA (D_4 99.7%), $^{13}C_1$-1,2-dihydroxybenzene ($_{13}C_1$ 99%), and D_5-S-BMA (D_5 99.1%) were obtained from C-D-N Isotope Inc. (Montreal, Quebec, Canada). D_5-S-PMA potassium (purity, no data) was purchased from Synthese Aptochem Inc. (Montreal, Quebec, Canada). Details of the analytical and quantification procedures have been previously described. The urinary creatinine concentrations were determined using a Hitachi 7600-110 automatic biochemistry analyzer (Hitachi, Japan).

Table

Table S1. Recoveries, method detection limits (MDLs) and relative standard deviation of BTEX in atmospheric samples

	Recovery(%)	MDL(μ g m^{-3})	RSD(%)
Benzene	75.2	0.24	2.38
Toluene	87.0	0.34	6.12
ethyl-benzene	94.9	0.36	4.8
m-Xylene	102.3	0.54	3.26
p-Xylene	97.4	0.54	3.26
o-Xylene	91.9	0.3	4.11
